# Cone letter charts: rapid color test using a range of letter sizes

**DOI:** 10.1007/s00417-023-06111-3

**Published:** 2023-05-26

**Authors:** Jeff Rabin, Erica Poole, Kiana Hall, William Price

**Affiliations:** University of the Incarnate Word Rosenberg School of Optometry, 9725 Datapoint Drive, San Antonio, TX 78229 USA

**Keywords:** Color vision, Visual acuity, Contrast sensitivity, Ocular disease


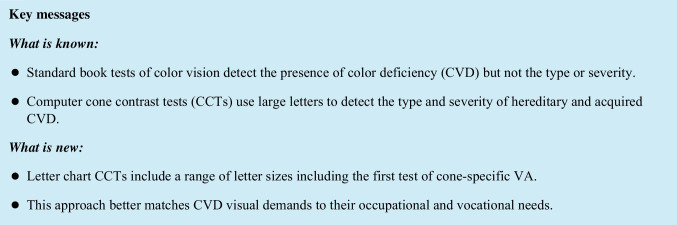


Normal color vision (CVN) allows recognition of numerous surface colors, precise wavelength discrimination, and color information essential for optimal safety and performance in myriad settings [1]. CVN derives from three cones: blue (short wavelength, S), green (middle wavelength, M), and red (long wavelength, L). Hereditary color vision deficiency (CVD; 8% males, 1/200 females) is as follows: L cones absent (protanopia, 1% of males); M cones absent (deuteranopia, 1%); and shift in cone sensitivity (protanomaly: L shifted toward M, 1%; deuteranomaly: M shifted toward L, 5%) [1, 2]. Acquired CVD occurs in many eye diseases [3, 4] and can be a biomarker for cognitive impairment [5]. Cone contrast sensitivity (CS) quantifies *type and severity* of CVD using large letters [2–5]. We expanded cone CS with more letter sizes (cone visual acuity, VA; small letter CS). A composite score was developed to quantify color vision across a range of letter sizes to improve matching of hereditary and acquired CVD abilities to visual demands. Cone-specific (L, M, S) CS functions were derived for potential research and clinical applications.

Computer-generated VA and CS charts were displayed on a calibrated Microsoft Surface computer presenting letters visible only to L, M, or S cones based on measurement of CIE chromaticity and luminance (CS-100 colorimeter, Konica Minolta) followed by transformation to cone excitations and cone contrasts [2, 3]. VA charts were as follows: 0.1 logMAR/row, five letters/row, and 0.02 logMAR/letter read correctly. L and M cone (LM) charts were as follows: 20/190 to 20/15 (0.98 to − 0.12 logMAR), 8% contrast. S cone charts were as follows: 20/240 to 20/50 (1.08 to 0.38 logMAR) and 64% contrast (contrasts derived from modified Innova Systems, Inc. test; larger and higher contrast letters were used for S cones due to their sparse retinal distribution) [2, 3]. CS charts were as follows: 10 rows, 5 letters/row, and logCS decreased 0.15/row yielding 0.03 logCS per letter correct. LM contrast range was as follows: 16–1%, S: 128–8%. Large letter CS was as follows: LM, 20/324, S, 20/430 [2, 3]. Small letter CS was as follows: LM 20/68, S 20/180. Testing was binocular with habitual correction in a dark room at 91.44 cm. Data were distributed normally (Jarque–Bera test). ANOVA and paired and unpaired *t*-tests with Bonferroni correction and regression analyses were used for descriptive and comparative analyses (Microsoft Excel version 2211). Fifteen CVNs (27 ± 6 YO) and 8 CVDs (32 ± 17), confirmed as CVN or CVD by Ishihara and anomaloscope testing, participated after written informed consent in accord with our IRB approved protocol.

**Fig. 1:**
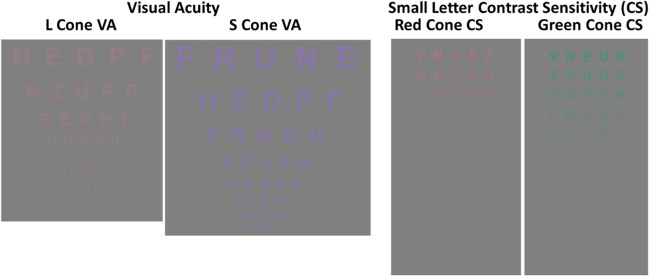
L cone- and S cone-specific VA charts (left panels) and small letter CS charts (right panels) as seen on the surface display. Letters were visible only to the specified cone type (please see text for further details). L and M cone VA charts were displayed at 8% contrast (color normals initially tested at 16% had scores corrected to 8% by adding 0.1 logMAR, S cone VA charts displayed at 64% contrast). The right panels show L and M cone small letter CS charts with contrast varying from 16 to 1% contrast for both small and large letter CS (please see text for additional details). Three versions of each test were used to discourage learning effects

**Fig. 2:**
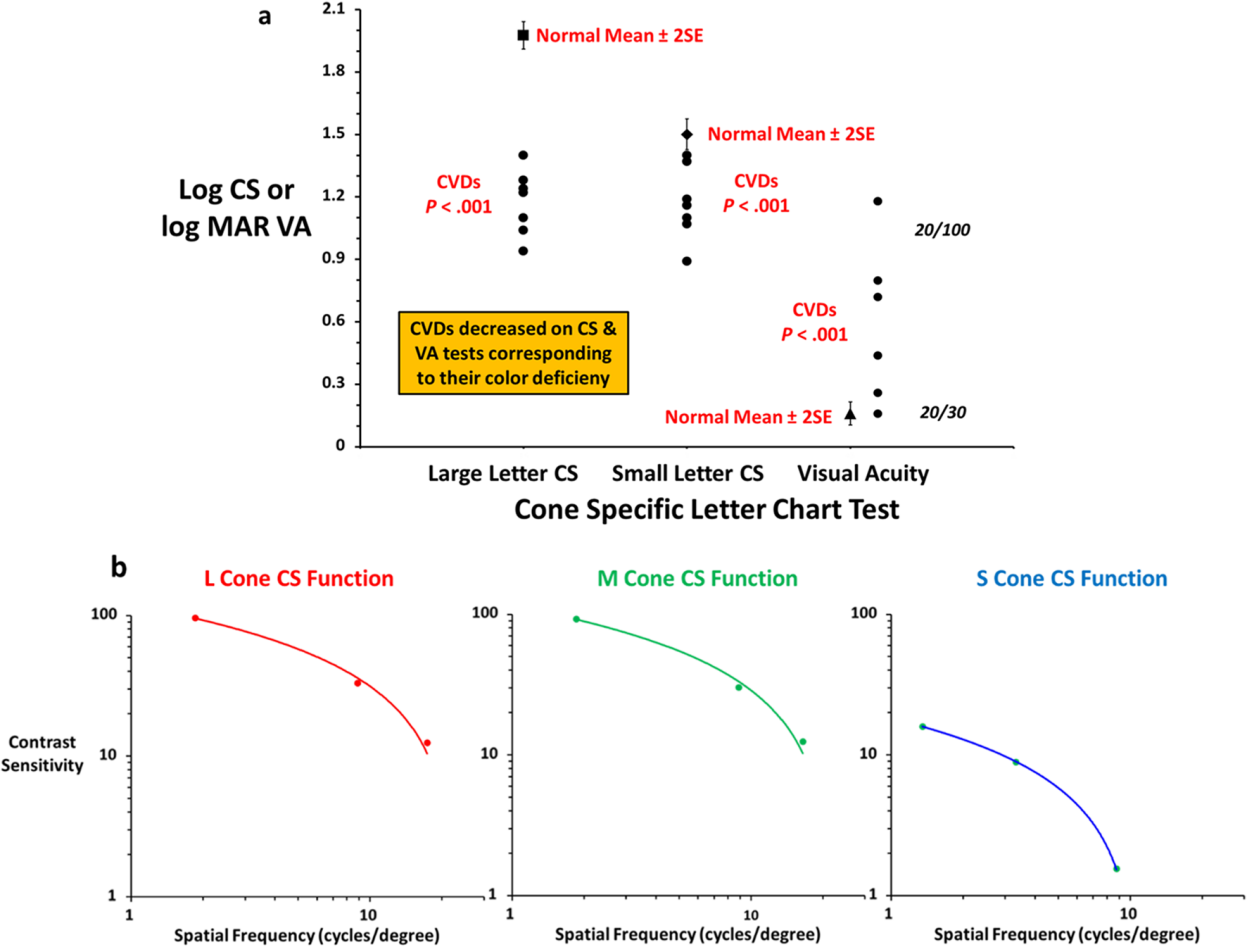
**a** Mean VA (logMAR) and large and small letter CS (logCS) are plotted for CVN subjects (mean ± 2SE, *n* = 15) with individual results shown for each CVD for their *color defective cone type*. Mean comparisons were highly significant for each test (*P* < .001, please see text for further details). **b** L, M, and S cone-specific CS functions derived from the best fit of CVD means across spatial frequency and contrast. The *Y*-axes are contrast sensitivity (1/contrast threshold), and the *X*-axes are spatial frequency (cycles/degree) with data plotted on absolute scales showing differences between L, M, and S cones. Please see text for additional comment and relevance

Figure 1 shows cone VA and CS charts (details in figure caption). Figure 2a shows CVN means (± 2SE) for each test with individual results from CVDs for their defective cone type (5 deutans, 3 protans). Mean differences between CVNs and CVDs were significant on each test (*P* < 0.001). A composite score based on sum of log scores for each test (VA corrected for sign) revealed a significant difference between CVNs (mean 3.3) vs. CVDs (mean 1.6; mean difference = 1.7, 95% CI = 1.25–2.21, *P* < 0.001). Cone CS functions were derived by converting mean CVN values to CS vs. spatial frequency and applying best-fit logarithmic regression (Fig. 2b).

Computer-generated cone-specific VA and CS charts offer a new, rapid clinical method to quantify color vision. Color tasks requiring fine detail (electronics, signal light detection, seeing details with macular dystrophies, AMD) may benefit more from cone VA and/or small letter CS. CVD physicians discriminating skin tones, and patients with acquired CVD from glaucoma or MS, may benefit more from large letter CS. Composite scores provide a comprehensive metric which may, in some cases, exceed sensitivity of current cone CS tests. Cone-specific contrast sensitivity functions derived herein exemplify absolute differences between cones offering detailed information potentially useful for both research and clinical applications.
